# Prevalence and indoor environment risk factors of parent-reported food allergy symptoms among preschool children in Haikou City, China: a cross-sectional survey

**DOI:** 10.3389/fpubh.2026.1699833

**Published:** 2026-04-22

**Authors:** Shoumin Wang, Qisheng Wu, Yifan Hu, Binxian Zhou, Shiheng Fan, Zhen Yan, Dee Yu, Jing Zhang

**Affiliations:** 1 Key Laboratory of Tropical Translational Medicine of Ministry of Education, School of Public Health, Hainan Academy of Medical Sciences, Hainan Medical University, Haikou, Hainan, China; 2 Wenchang Center for Disease Control and Prevention, Wenchang, Hainan, China

**Keywords:** food allergy, indoor environment, inventory survey, preschool children, tropical zone

## Abstract

**Objective:**

To understand the prevalence of parent-reported food allergy symptoms among preschool children in Haikou City and investigate indoor environmental risk factors for it.

**Methods:**

A cross-sectional survey was conducted with 3,049 preschool children across eight kindergartens in Haikou City from December 2020 to January 2021. The survey aimed to analyze the influence of indoor environmental factors on parent-reported food allergy symptoms in these children. Data collection was facilitated using EpiData 3.1, while SPSS 25.0 was employed for data analysis. The Chi-square (*χ*^2^) test was applied for univariate analysis, and multivariate logistic regression was utilized to examine the indoor environmental factors affecting parent-reported food allergy symptoms in children. A *p*-value of less than 0.05 was considered to indicate statistical significance.

**Results:**

The prevalence of parent-reported food allergy symptoms in preschool children was 9.25%. Multivariate unconditional logistic regression results suggested that age (odds ratio (OR) = 1.891, 95% confidence interval (CI): 1.246–2.871), nationality other than the Han (OR = 1.753, 95%CI: 1.097–2.802), mother’s education level junior college and above than below junior college (OR = 1.395, 95%CI: 1.051–1.851), family allergy history (OR = 2.893, 95%CI: 2.233–3.748), the only child (OR = 1.351, 95%CI: 1.031–1.770), furniture purchased in child’s first year (OR = 1.774, 95%CI: 1.263–2.493), mould on surfaces during pregnancy (OR = 1.540, 95%CI: 1.025–2.313), damp exposure during pregnancy (OR = 1.678, 95%CI: 1.120–2.515), exposure to passive smoking (OR = 1.483, 95%CI: 1.143–1.925) were risk factors for parent-reported food allergy symptoms.

**Conclusion:**

This study highlights the significant role of the indoor home environment, alongside genetic factors, in the development of parent-reported food allergy symptoms in children. Implementing strategies to reduce indoor moisture and avoid tobacco smoke exposure is essential for preventing parent-reported food allergy symptoms in preschool-aged children.

## Background

Allergic diseases are typically mediated by serum-specific immunoglobulin E (sIgE), with common conditions including asthma, allergic rhinitis, atopic dermatitis, and food allergy. Being the most prevalent chronic non-infectious disease in children, many can develop symptoms within the first year of life. Over the past 20 years, the prevalence of food allergy has significantly increased (1), currently affecting nearly 8% of children and 5% of adults worldwide (2). Food allergy can be broadly categorized into immune responses mediated by specific IgE and non-IgE-mediated activation of effector cells (3). Reports from some Western countries indicate that the prevalence of food allergy in children aged 0–1 year exceeds 10% (4). In Asia, the prevalence of food allergy in children stands at 4–5% in Singapore, 10.9% in South Korea, and 12.6% in Japan (5), with a 10% prevalence in preschool children (6). A study by the Capital Institute of Pediatrics, covering children aged 0–14 years across 31 provinces and cities nationwide, found a 5.83% prevalence of food allergy, with the highest rates in East China (7.38%) and Northeast China (7.38%), and the lowest in Northwest China (4.35%). Several risk factors are linked to the onset of atopic diseases, such as family history of allergy (7), feeding practices (8, 9), dietary habits (10), antibiotic usage (11), mode of delivery (12), and air pollution (13). In early stages, children’s immune systems are not fully developed, making them susceptible to allergic reactions (14). According to the survey, compared to after birth (postnatal), families who redecorated their homes less frequently during the mother’s pregnancy (prenatal) had less new furniture, less house renovations, less ventilation in the house, more condensation in the Windows in winter, and a higher prevalence of food allergies in children with more mould/moisture. Children exposed to new furniture, house finishes, window condensation, and mould/damp environments with higher levels of indoor air pollution had higher rates of FA (15). It can be seen that the indoor environment has an important impact on preschool children. In summary, the primary factors influencing allergic diseases include genetic and environmental factors, with genetic factors unlikely to account for a rapid increase in prevalence over a short period. Furthermore, since children’s immune systems are not fully developed, they are more vulnerable to environmental influences than adults (16). Therefore, this study aims to examine the impact of indoor environmental factors on food allergy in preschool children and explore the relationship between food allergy and the indoor environment in Haikou City, to provide insights into the prevention and treatment of food allergy in this demographic.

## Method

### Subjects

Multistage stratified cluster sampling method was used to conduct the survey. Eight kindergartens were randomly selected from four administrative districts (Xiuying District, Longhua District, Qiongshan District, Meilan District) in Haikou City, and all children in the kindergartens were investigated. The parents of all children in each kindergarten were invited to participate in the questionnaire survey. Questionnaires were distributed 4,248 times, and questionnaires were collected 3,643 times, with a response rate of 85.76%. The survey was conducted in January 2021. The parents of all children surveyed gave informed consent and signed the informed consent form.

### Questionnaire

Specific distribution method of the questionnaire: before the survey, we contacted Haikou Health Education Institute and the principals of kindergartens. First, we gave kindergarten school doctors and class teachers a unified professional training. We sent the questionnaires to the kindergarten, and the school doctors distributed them to the class teachers, who distributed them to the parents. The parents could take the questionnaires home and complete them within a week and return them to the class teacher. Then, all the teachers in the classes would fill in the questionnaires and give them to the school doctor, who collected all the kindergarten classes’ filled questionnaires and gave them to Haikou Health Education Institute. The questionnaire includes demographic characteristics, child allergies, and residential indoor environmental factors.

The questionnaire was prepared in accordance with the ALLHOME-2 in Naydenov’s doctoral thesis and the questionnaire used by Bornehag in the Dampness in Buildings and Health survey study (17, 18), with minor revisions based on specific conditions in China and Haikou. The primary outcome was parent-reported food allergy symptoms, ascertained via a single question regarding adverse reactions to food. We did not perform confirmatory oral food challenges, specific IgE testing, or skin prick tests. The survey had the following six components:

(1) Demographic characteristics: including children’s gender, age, height/weight at birth and at present, and children’s kindergarten enrollment; family allergy history, duration of breastfeeding, maternal pregnancy cycle, maternal age and occupation during pregnancy and so on.
(2) Child allergies: asthma, rhinitis, eczema, food allergy and related symptoms.
(3) Residential indoor environmental factors:

① Architectural characteristics: including building location and age, floor and area of residence, window frame type, number of glass layers, and external environment of buildings (whether adjacent to traffic arteries, rivers/lakes, commercial areas and industrial areas).

② Decoration and furniture: including floor materials in children’s rooms, wall materials in children’s rooms, whether new furniture is purchased, and whether redecoration is done and so on.

③ Air conditioning use and ventilation: including whether to use air conditioning and its frequency, ventilation mode (kitchen range hood, kitchen exhaust fan and bathroom exhaust fan).

④ Building moisture: including moldy phenomenon, moisture phenomenon, water damage, condensed water in windows, damp clothes and bedding, and moldy smell and so on.

⑤ Odor perception is the self-report of indoor personnel’s odor, such as the stale odor, irritating odor and tobacco odor caused by poor ventilation.

⑥ Living habits include window opening habits, indoor smoking, bedding drying and children’s room cleaning.

⑦ Indoor animals include pet breeding, avoidance behavior (avoidance behavior 1: giving up keeping pets due to allergic diseases in the family, avoidance behavior 2: not keeping pets due to allergic diseases in the family) and indoor animals such as flies, mosquitoes, cockroaches, mice, dust mites and so on.

⑧ Indoor equipment and supplies include printers, copiers, dehumidifiers, air purification equipment, ion generators, mosquito repellents, incense and so on.

The questionnaire of this survey was mainly based on parents’ reported food allergy. The corresponding questions in the questionnaire are “Has your child ever had allergic symptoms such as eczema, hives, diarrhea, swollen lips or eyes caused by foods?”, If the answer is “yes,” the surveyed children are considered to have food allergies. Therefore, the food allergies in this survey included both IgE mediated and non-IGE mediated, because the reporters were parents or people living with the surveyed children and they could not identify the specific types of food allergies.

The sample size of cross-sectional study is calculated as follows:

$N=Z\alpha^{2}\times \mathrm{pq}/d^{2}$

p is the expected prevalence rate, q = 1−p, d is the allowable error, Zα is the statistic of the significance test, and *N* is the sample size.

The factors that determine the sample size of cross-sectional studies come from several aspects: (1) the expected prevalence rate (p); (2) The requirement for the accuracy of the survey results, that is, the larger the allowable error (d), the smaller the sample size required; (3) Significance level (*α*), the smaller the *α* value, the higher the significance level and the larger the sample size requirements.

According to the data, the distribution rate of at least one allergic disease in all children of concern is 50%, the sampling error is 2.5%, and the confidence level is 95%.

$N=1.962^{*}\;0.5^{*}\;0.5/0.0252\;\text{material}\;1,600$

Since this study is a cluster sampling, the sample size needs to be expanded by 1.5 times: 1,600 × 1.5 = 2,400.

### Statistical analyses

Data were compiled using EpiData version 3.1 and analyzed with SPSS version 25.0. The Chi-square (*χ*^2^) test was employed for univariate analysis, while multivariate logistic regression analysis was utilized to examine the indoor factors influencing food allergy prevalence in children. A *p*-value of less than 0.05 was deemed to indicate statistical significance.

## Results

### Prevalence of food allergy in preschool children in Haikou City

**Figure 1:**
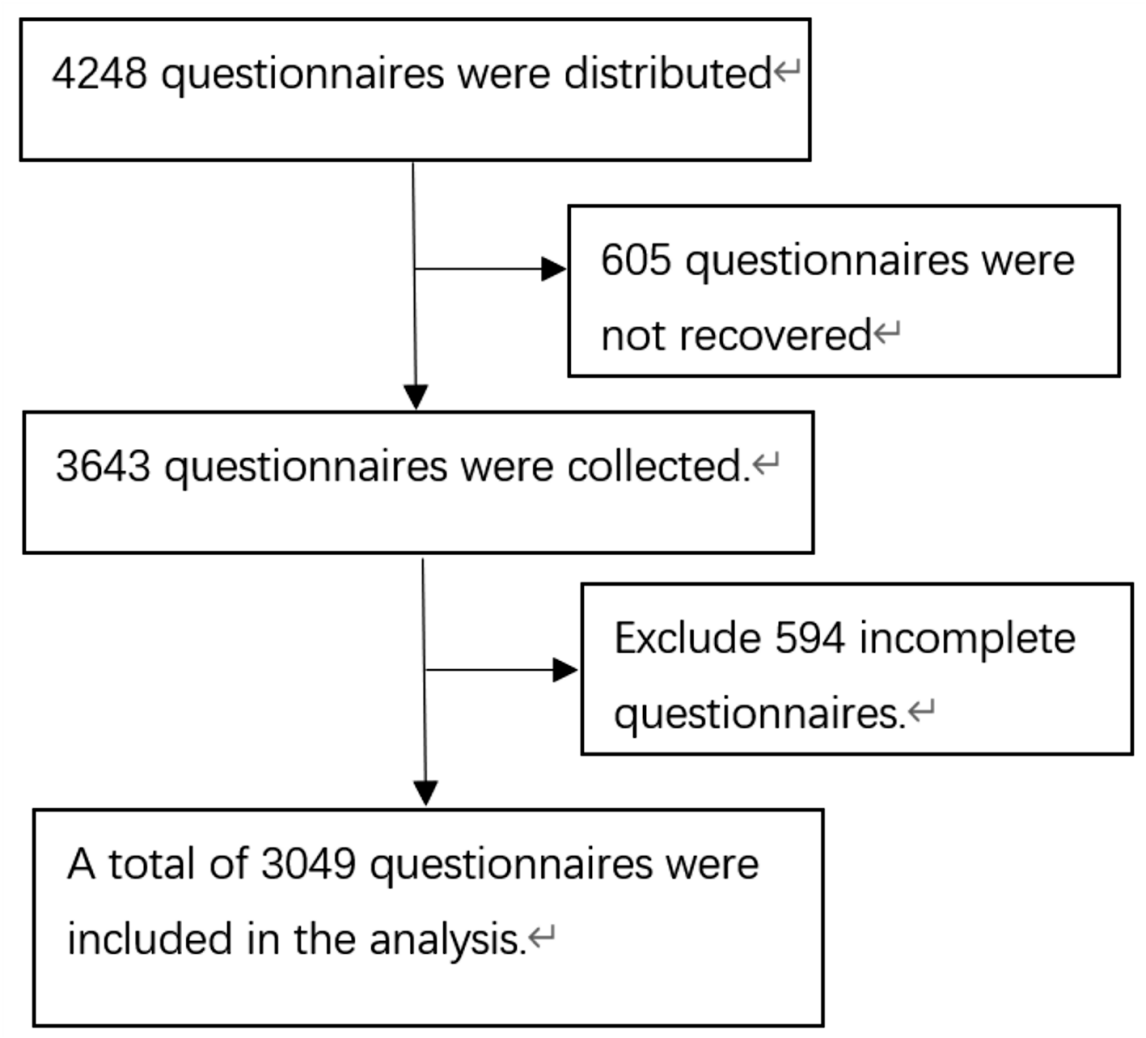
Flow chart of participants.

The research group conducted a questionnaire survey on selected kindergarten children from December 2020 to January 2021. A total of 4,248 questionnaires were distributed, and 3,643 questionnaires were returned, with a recovery rate of 85.76%. After excluding questionnaires with incomplete answers, 3,049 valid questionnaires were collected. The specific flow chart is shown in Figure 1. Among the 3,049 preschool children participating in the survey, the prevalence of food allergy was 9.25%. The *χ*^2^ analysis indicated statistically significant associations between the prevalence of food allergy in preschool children in Haikou City and several demographic factors, including nationality, age, mother’s education level, father’s education level, family history of allergy, age of starting group childcare, and the only child (*p* < 0.05) see Table 1.

**Table 1:** Comparison of food allergy among preschool children with different characteristics in Haikou (*n* = 3,049).

Characteristics	Number of respondents (*n* = 3,049)	Number of food allergy cases (*n* = 282)	Detection rate (%)	*χ* ^2^	*p* value
Nationality				7.241	0.007
Han	2,884	257	8.9		
Other	165	25	15.2		
Gender				0.464	0.496
Boys	1,693	162	9.6		
Girls	1,356	120	8.8		
Age				8.909	0.031
3 years old	695	53	7.6		
4 years old	1,145	97	8.5		
5 years old	793	80	10.1		
6 years old	416	52	12.5		
Mode of birth				0.887	0.346
Natural birth	1,970	175	8.9		
Cesarean section	1,079	107	9.9		
Breast-feeding				1.789	0.181
<6 months	1,260	106	8.4		
≥6 months	1,789	176	9.8		
Mother’s education level				17.128	<0.001
Below college or university	1,272	85	6.7		
College or university and above	1,777	197	11.1		
Father’s education level				10.824	0.001
Below junior college	1,163	82	7.1		
Junior college and above	1,886	200	10.6		
Family allergy history				100.232	<0.001
Yes	985	166	16.9		
No	2,064	116	5.6		
Start age for group care				5.127	0.024
<3 years old	882	98	11.1		
≥3 years old	2,167	184	8.5		
The only child				10.080	0.001
Yes	866	103	11.9		
No	2,183	179	8.2		

### Univariate analysis of food allergy with indoor environmental variables

The analyses revealed that type of residence, nighttime summer ventilation, furniture purchased pre-pregnancy, home renovations pre-pregnancy, furniture purchased during pregnancy, home renovations during pregnancy, furniture purchased in child’s first year, home renovations in child’s first year, furniture purchased during the past 12 months, mould on surfaces pre-pregnancy, damp exposure pre-pregnancy, mould on surfaces during pregnancy, damp exposure during pregnancy, mould on surfaces in child’s first year, damp exposure in child’s first year, mould on surfaces in the last 12 months, damp exposure in the last 12 months, pet ownership, cleaning frequency, musty odor presence, tobacco odor presence, perception of dry air, perception of humid air, exposure to passive smoking, were the main environmental factors influencing the prevalence of children’s food allergy (*p* < 0.05). See Table 2.

**Table 2:** Univariate analysis of food allergy and indoor environmental variables (*n* = 3,049).

Factors	Number of individuals	Number of cases	%	*χ* ^2^	*p* value
Type of residence				13.747	0.001
Apartment	1,846	199	10.8		
Villa	506	39	7.7		
Other	697	44	6.3		
Nighttime summer ventilation				5.828	0.016
No	217	30	13.8		
Yes	2,832	252	8.9		
Winter heating				0.875	0.350
No	2,234	200	9.0		
Yes	815	82	10.1		
Furniture purchased pre-pregnancy				15.950	<0.001
No	2,566	214	8.3		
Yes	483	68	14.1		
Home renovations pre-pregnancy				10.012	0.002
No	2,841	250	8.8		
Yes	208	32	15.4		
Furniture purchased during pregnancy				14.238	<0.001
No	2,780	240	8.6		
Yes	269	42	15.6		
Home renovations during pregnancy				7.509	0.006
No	2,910	260	8.9		
Yes	139	22	15.8		
Furniture purchased in child’s first year				25.814	<0.001
No	2,733	228	8.3		
Yes	316	54	17.1		
Home renovations in child’s first year				21.725	<0.001
No	2,899	252	8.7		
Yes	150	30	20.0		
Home renovations during the past 12 months				8.248	0.004
No	2,831	250	8.8		
Yes	218	32	14.7		
Mould on surfaces pre-pregnancy				24.758	<0.001
No	2,721	227	8.3		
Yes	328	55	16.8		
Damp exposure pre-pregnancy				28.090	<0.001
No	2,749	229	8.3		
Yes	300	53	17.7		
Mould on surfaces during pregnancy				30.393	<0.001
No	2,794	234	8.4		
Yes	255	48	18.8		
Damp exposure during pregnancy				35.521	<0.012
No	2,801	233	8.3		
Yes	248	49	19.8		
Mould on surfaces in child’s first year				21.456	<0.001
No	2,787	237	8.5		
Yes	262	42	17.2		
Damp exposure in child’s first year				22.997	<0.001
No	2,823	241	8.5		
Yes	226	41	18.1		
Mould on surfaces in the last 12 months				8.672	0.003
No	2,808	247	8.8		
Yes	241	35	14.5		
Damp exposure in the last 12 months				13.625	<0.001
No	2,842	248	8.7		
Yes	207	34	16.4		
Pet ownership				12.205	<0.001
No	2,718	234	8.6		
Yes	331	48	14.5		
Cleaning frequency				15.356	0.002
About 1 time/day	1,015	71	7.0		
About 2–3 times/week	1,288	118	9.2		
About 1 time/week	642	80	12.5		
Less than 1 time/week	104	13	12.5		
Kitchen ventilation				0.209	0.647
No	89	7	7.9		
Yes	2,960	275	9.3		
Bathroom ventilation				2.068	0.150
No	125	7	5.6		
Yes	2,924	275	9.4		
Musty odor presence				9.370	0.002
No	2,778	243	8.7		
Yes	271	39	14.4		
Tobacco odor presence				3.859	0.049
No	2,750	245	8.9		
Yes	299	37	12.4		
Perception of dry air				8.235	0.004
No	2,191	182	8.3		
Yes	858	100	11.7		
Perception of humid air				10.933	0.001
No	1,899	150	7.9		
Yes	1,150	132	11.5		
Exposure to passive smoking				13.215	<0.001
No	2,077	165	7.9		
Yes	972	117	12.0		

### Multivariate analysis of food allergy and indoor environmental variables

Food allergy was used as the dependent variable (0 = no; 1 = yes), variables significant in univariate analysis were introduced into the logistic regression model, and 9 variables from the final entry main effect model were associated with the prevalence of food allergy. Among these, age (OR = 1.891, 95%CI: 1.246–2.871), nationality other than the Han (OR = 1.753, 95%CI: 1.097–2.802), mother’s education level junior college and above than below junior college (OR = 1.395, 95%CI: 1.051–1.851), family allergy history (OR = 2.893, 95%CI: 2.233–3.748), the only child (OR = 1.351, 95%CI: 1.031–1.770), furniture purchased in child’s first year (OR = 1.774, 95%CI: 1.263–2.493), mould on surfaces during pregnancy (OR = 1.540, 95%CI: 1.025–2.313), damp exposure during pregnancy (OR = 1.678, 95%CI: 1.120–2.515), exposure to passive smoking (OR = 1.483, 95%CI: 1.143–1.925) were risk factors for food allergy. See Table 3.

**Table 3:** Multivariate logistic regression analysis of risk factors for food allergy among preschool children in Haikou (*n* = 3,049).

Factors	Level	*β*	SE	Wald *χ*^2^	*p* value	OR	95% CI
Age	3 years old					1.000	
	6 years old	0.637	0.213	8.946	0.003	1.891	1.246–2.871
Nationality	Han					1.000	
	Other	0.561	0.239	5.501	0.019	1.753	1.097–2.802
Mother’s education level	Below junior college					1.000	
	Junior college and above	0.333	0.144	5.322	0.021	1.395	1.051–1.851
Family allergy history	No					1.000	
	Yes	1.062	0.132	64.655	<0.001	2.893	2.233–3.748
The only child	No					1.000	
	Yes	0.301	0.138	4.751	0.029	1.351	1.031–1.770
Furniture purchased in child’s first year	No					1.000	
	Yes	0.573	0.173	10.931	0.001	1.774	1.263–2.493
Mould on surfaces during pregnancy	No					1.000	
	Yes	0.432	0.208	4.324	0.038	1.540	1.025–2.313
Damp exposure during pregnancy	No					1.000	
	Yes	0.518	0.206	6.294	0.012	1.678	1.120–2.515
Exposure to passive smoking	No					1.000	
	Yes	0.394	0.133	8.779	0.003	1.483	1.143–1.925

## Discussion

Previous research has demonstrated that a combination of genetic, developmental, and environmental factors plays a critical role in modulating the immune system and predisposing individuals to various diseases (19, 20). In this context, our study utilized a cross-sectional survey approach to assess the prevalence of parent-reported food allergy symptoms among preschool children in Haikou City, alongside identifying key indoor environmental factors that may contribute to these allergies. The goal was to elucidate the primary factors influencing parent-reported food allergy symptoms in this demographic, thereby laying a foundation for the development of targeted early intervention strategies.

The survey revealed a 9.25% prevalence rate of food allergy among preschool children in Haikou City. This figure stands in contrast to the higher rates observed in other regions; for instance, a study reported a food allergy prevalence of 11.52% (21)in children aged 2–7 years in Urumqi in 2019, and an even higher rate of 16.8% (22) among preschoolers aged 3–6 years in Shanghai. These discrepancies highlight significant regional variations in the prevalence of food allergy.

The reason why 16.3% of the data were not included in the data analysis: they did not meet the inclusion criteria of this survey. The research object of this survey was preschool children aged 3–6 years old, and some of the excluded data were non-preschool children.

The findings from our multivariate logistic regression analysis align with existing literature, identifying family history of allergies as a significant risk factor for parent-reported food allergy symptoms among preschool children in Haikou City. This correlation underscores the pivotal role of genetic predispositions in the development of food allergy. Furthermore, our study echoes the findings of prior research by highlighting that children from families with a history of allergies are at an increased risk of developing allergic conditions (23). Intriguingly, we also discovered that a higher level of maternal education constitutes a risk factor for food allergy in preschoolers, a conclusion supported by numerous scholars (24). Higher maternal education may be associated with greater health literacy and heightened awareness of allergic symptoms, leading to increased reporting rather than increased incidence. Alternatively, educated parents may have better access to healthcare and thus receive diagnoses more frequently. Future studies should include objective measures of specific cleaning behaviors and healthcare-seeking patterns to disentangle these mechanisms. Such reduced exposure potentially hampers the proper maturation of the immune system, making it less adept at tolerating allergens (25).

Beyond genetic predispositions, environmental factors significantly contribute to the development of food allergy in children (26). Our study provides direct epidemiological evidence for this paradigm by identifying several modifiable indoor environmental exposures during the prenatal and early-life periods as independent risk factors for food allergy in preschoolers. Specifically, we found that exposure to mould and dampness during pregnancy, purchasing new furniture in the child’s first year of life, and exposure to passive smoking were all significantly associated with increased food allergy risk. Critically, these factors often co-occur, forming a “syndrome of adverse indoor environmental exposures” that may exert synergistic effects on the developing immune system.

The convergence of these exposures during critical developmental windows suggests plausible biological pathways underlying our observed associations, fitting within the Developmental Origins of Health and Disease (DOHaD) framework (27). Prenatal exposure to mould components, environmental tobacco smoke, and certain VOCs can act as exogenous danger signals. They may activate maternal and placental innate immunity, leading to a systemic pro-inflammatory state that can skew fetal immune development toward a Th2-dominant phenotype, which is central to allergic sensitization (28). Dampness and mould are associated with altered indoor microbial ecology, which can influence the maternal and infant microbiome—a key educator of immune tolerance. Notably, this path mechanism is supported by the work of Lu et al., who documented that exposure to tobacco smoke and renovation-related VOCs (e.g., formaldehyde) compromises epithelial barrier integrity, thereby increasing susceptibility to food allergens (29). A compromised barrier facilitates the abnormal presentation of food antigens, promoting sensitization. Compounds such as phthalates (acting as endocrine disruptors) and constituents of tobacco smoke are known to induce epigenetic modifications (e.g., DNA methylation) in genes governing immune regulation (30). Such programming alterations during sensitive periods may have long-lasting effects on allergic predisposition.

Therefore, our results substantiate the DOHaD hypothesis by highlighting the prenatal and early postnatal indoor environment as a programmable interface shaping long-term allergy risk. This shifts the perspective from viewing these exposures merely as isolated hazards to recognizing them as interconnected modifiable targets for primary prevention. Public health strategies aimed at improving indoor air quality—through humidity control, smoke-free homes, and the use of low-emission materials—especially during pregnancy and infancy, could be pivotal in mitigating the burgeoning burden of food allergy.

This study was conducted in Haikou City, Hainan Province, China. Haikou City has a large climate gap with the inland areas of China, with high temperature and humidity throughout the year and higher air quality than the inland areas, which can effectively reflect the relationship between food allergy and indoor environmental factors of preschool children in tropical areas.

Most modern people spend more than 90% of their time indoors, especially preschool children, who spend 80 to 90% of their time at home and in kindergarten. Although the epidemic will have an impact on people, the impact on children is less than that on adults. Moreover, this survey focuses on the relationship between the indoor environment and food allergies of preschool children, and generally speaking, the impact is not large (31).

Despite the insightful findings, this study has several limitations that warrant consideration. First, the outcome relied entirely on parental self-report of symptoms in response to a single question. Unlike the ‘gold standard’ of double-blind placebo-controlled food challenges or even physician assessment based on specific IgE levels and skin prick tests, this subjective measure is highly susceptible to misclassification bias. Second, this approach inevitably conflates distinct clinical entities. We cannot distinguish between IgE-mediated food allergy (which carries a risk of anaphylaxis), non-IgE mediated allergy (such as food protein-induced enterocolitis), and non-immune mediated food intolerances (such as enzyme deficiencies or irritant effects). While the parent may perceive all of these as an ‘allergy,’ their pathophysiology, management, and prognosis differ substantially. Third, the use of a single question likely overestimates the true burden of disease. Therefore, our reported prevalence should be interpreted as the prevalence of parent-perceived food hypersensitivity, rather than confirmed immunological allergy. Future studies should incorporate clinical validation to improve specificity. Then, the cross-sectional design limits our ability to infer causality between indoor environmental factors and the prevalence of food allergy among preschool children. Longitudinal studies are needed to establish temporal relationships and causality, and the reliance on parental reporting for the diagnosis of food allergy and environmental exposures may introduce recall bias, potentially affecting the accuracy of the data collected. The study focused on a specific geographic area, which may limit the generalizability of the findings to other regions with different environmental conditions and lifestyle practices. Lastly, the study did not account for all possible confounding factors, such as diet and outdoor environmental exposures, which could also influence the development of food allergies in children.

In China, ethnicity is frequently a proxy for geographic region, dietary patterns, and socioeconomic conditions. Minority populations may have distinct weaning practices, higher rates of specific food exposures, or different levels of access to diagnostic services. Furthermore, language or cultural barriers in the survey instrument may have influenced interpretation of the question regarding adverse reactions. Without detailed data on household income, parental occupation, dietary diversity, and geographic location, residual confounding cannot be ruled out. This association should be explored in studies designed specifically to examine dietary and environmental exposures across ethnic groups.

In conclusion, In this cohort, the prevalence of parent-reported adverse reactions to food was 9.25%. This likely represents an overestimate of the true prevalence of physician-diagnosed IgE-mediated food allergy, given the lack of objective clinical confirmation and the probable inclusion of food intolerances. And our study underscores that, alongside genetic predispositions, the indoor home environment plays a pivotal role in the development of food allergy in children. Measures aimed at reducing indoor moisture and avoiding exposure to tobacco smoke are crucial for the prevention of food allergy in preschool children. Nonetheless, given the cross-sectional nature of this survey, the findings might be subject to certain limitations. For a more comprehensive understanding of the factors influencing food allergy among preschool children in Haikou City, further research employing case–control or cohort study designs is warranted.

## Data Availability

The data analyzed in this study is subject to the following licenses/restrictions: the datasets generated and/or analysed during the current study are no publicly available due the data belongs to the School of Public Health of Fudan University but are available from the corresponding author on reasonable request. Requests to access these datasets should be directed to Qisheng Wu, 1414200866@qq.com.
